# Long-Term Statistical Process Monitoring of an Ultrafiltration Water Treatment Process

**DOI:** 10.1021/acsestengg.4c00042

**Published:** 2024-05-31

**Authors:** Taylor R. Grimm, Amos Branch, Kyle A. Thompson, Andrew Salveson, John Zhao, Darrell Johnson, Amanda S. Hering, Kathryn B. Newhart

**Affiliations:** † Department of Statistical Science, Baylor University, Waco, Texas 76798, United States; ‡ Carollo Engineers, Inc., Walnut Creek, California 94598, United States; § Las Virgenes Municipal Water District, Calabasas, California 91302, United States; ∥ Department of Geography and Environmental Engineering, United States Military Academy, West Point, New York 10996, United States

**Keywords:** statistical process monitoring, fault detection, principal component analysis, ultrafiltration, machine learning

## Abstract

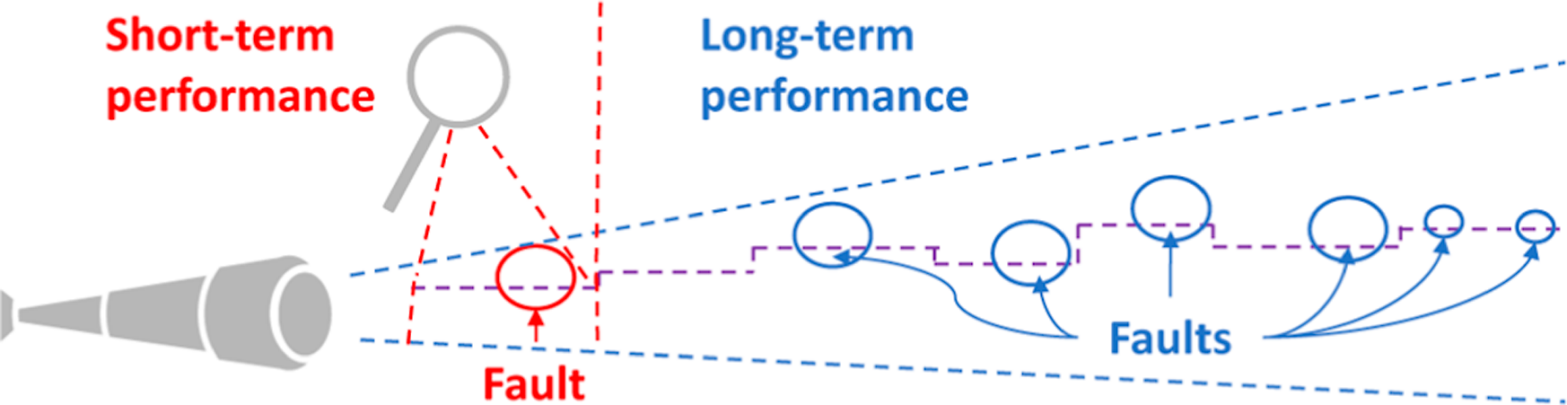

As water treatment technology has improved, the amount of available process data has substantially increased, making real-time, data-driven fault detection a reality. One shortcoming of the fault detection literature is that methods are usually evaluated by comparing their performance on hand-picked, short-term case studies, which yields no insight into long-term performance. In this work, we first evaluate multiple statistical and machine learning approaches for detrending process data. Then, we evaluate the performance of a PCA-based fault detection approach, applied to the detrended data, to monitor influent water quality, filtrate quality, and membrane fouling of an ultrafiltration membrane system for indirect potable reuse. Based on two short case studies, the adaptive lasso detrending method is selected, and the performance of the multivariate approach is evaluated over more than a year. The method is tested for different sets of three critical tuning parameters, and we find that for long-term, autonomous monitoring to be successful, these parameters should be carefully evaluated. However, in comparison with industry standards of simpler, univariate monitoring or daily pressure decay tests, multivariate monitoring produces substantial benefits in long-term testing.

## 1 Introduction

Advancements in water treatment include improved technologies, such as ultrafiltration (UF), and improved process monitoring, with the addition of instrumentation and advanced controls. The various sensors and analyzers in the treatment process have led to an increased availability of data, potentially allowing for better identification of faults in the process. However, large amounts of data can present a challenge for operators. Changes to the treatment process, such as UF membrane fouling or undesired changes in influent or effluent quality, must be monitored properly to maintain filtration efficiency and to avoid accidental discharge of potentially harmful contaminants. The primary method for process monitoring in water treatment is for operators to visually examine plotted trends or values of individual process variables to determine if they differ significantly from normal operating conditions. This method of manual fault detection can be especially difficult when changes are small, occur gradually over time, or affect multiple variables simultaneously. Furthermore, meaningful changes in the relationships between process variables can be overlooked entirely by a manual monitoring approach because such changes can be difficult or impossible to assess visually in real time or with a univariate monitoring strategy, such as setting fixed upper and lower control limits. To improve fault detection efficiency and performance, a fault detection approach capable of capturing changes between variables is needed.

Statistical process monitoring (SPM) is a data-driven approach to real-time fault detection that compares new, testing observations with fault-free observations collected during a previously observed training period. Unlike other data-driven fault detection approaches, such as classification-based methods,^1,2^ SPM requires no prior knowledge of the faults that may appear, and an SPM model can be built even if few or no faults have been previously observed. Prior knowledge of faults is unnecessary because SPM functions by identifying significant departures of testing observations from the normal operating behavior during the training period.^3,4^

A popular and easy-to-implement approach to fault detection with SPM is to apply univariate control charts on each variable of interest. A wide variety of univariate methods exist, such as Shewhart,^5,6^ cumulative sum (CUSUM),^7,8^ and exponentially weighted moving average (EWMA)^9,10^ control charts. A discussion of various univariate SPM methods is given by Woodall and Montgomery.^11^ Among these methods, the Shewhart control chart is the simplest and most interpretable. With Shewhart charts, a variable is considered in-control (IC) if the value is within a given number of standard deviations of the mean. If the value is outside of this range, it is considered out-of-control (OC).

The major drawback of univariate SPM methods is their ability to only detect abnormally high or low values of individual process variables. In some cases, the values of all process variables may be acceptable individually, but when they are considered jointly, they would be considered unusual. Multivariate SPM (MSPM) can detect abnormal changes in either the means or relationships among the variables, which are impossible to detect with univariate SPM methods.

Many methods for MSPM exist, including multivariate cumulative sum (MCUSUM),^12−14^ multivariate exponentially weighted moving average (MEWMA),^15,16^ and principal component analysis (PCA)-based^17−19^ control charts. A review and discussion of these and additional MSPM methods is given by Bersimis et al.^20^ In this research, we focus on PCA and modified versions of PCA because water treatment processes are commonly high-dimensional and noisy, both of which are easily handled with PCA-based monitoring methods.

A key aspect of our approach is to assign each process variable to one of two groups: monitoring or explanatory. Monitoring variables are the variables most directly related to process monitoring goals. Explanatory variables may affect or be related to changes in the monitoring variables but are not of primary interest in monitoring. In the context of water treatment, examples of monitoring variables are regulated water quality parameters, bulk surrogates used as treatment performance metrics, and operational variables directly indicative of process health. Examples of explanatory variables include controlled process variables or influent water quality variables whose values are measured for informational purposes but are not directly used for control and may correlate to other process changes.

Once the variables are classified, explanatory variables are used to detrend the monitoring variables. Detrending removes explainable or expected variability in the monitoring variables. As a result, observations are only flagged as OC when unexpected changes occur. To detrend monitoring variables, a statistical or machine learning (ML) model is fit to each monitoring variable using the explanatory variables as model predictors. Then, model residuals are obtained, which are the differences between the model predicted values and the actual values of the monitoring variable. Some work has been done using multiple regression to detrend monitoring variables prior to monitoring.^21,22^ It is also common to monitor the residuals from ML or time series models, but these methods do not distinguish between explanatory and monitoring variables in the process.^23−28^ Instead, they predict each monitoring variable using previous values or other process variables as predictors. Separating process variables into “explanatory” or “monitoring” reduces the number of variables being monitored directly and leads to a more straightforward interpretation of any OC signals as attributable to changes in the process that are not predicted by the explanatory variables.

Finally, an important factor to consider when comparing different fault detection approaches is the length of time of the study. Most literature focuses on short periods of time,^19,29−31^ often with hand-picked case studies of a few days or weeks.^32−36^ However, monitoring methods are implemented for real-time usage over long periods of time, and short-term performance may not always translate to long-term detection ability. Changes in influent quality, equipment, or operation are expected to occur over time and can affect model performance. To maintain long-term monitoring performance, adjustments to SPM and MSPM methods are needed. To adjust univariate SPM methods for long-term monitoring, a rolling training window can be used to automatically update upper and lower control limits to account for nonstationarity (e.g., seasonal changes in means). The size of the rolling window; the number of consecutive observations classified as OC needed to trigger an alarm; and the selection of control limits to achieve a prespecified number of false positives are unique to the process. These must be chosen to maintain low false positives and adapt to evolving conditions. MSPM PCA-based methods used without any adjustments are referred to as static PCA, but many adjustments to PCA have been proposed to improve fault detection performance.^37^ These include dynamic PCA,^19,38^ adaptive PCA,^39,40^ and adaptive-dynamic PCA (AD-PCA).^33,41,42^ Dynamic PCA accounts for autocorrelation in the data by including lags of detrended monitoring variables, adaptive PCA uses a rolling window approach to account for nonstationarity, and AD-PCA incorporates both adjustments, making it suited for monitoring multivariate autocorrelated data over long periods of time.

The purpose of this paper is to assess the fault detection performance of AD-PCA over a long period of time and to compare it to adaptive univariate Shewhart control charts. We begin by describing the data and methods used for fault detection in Section 2. The MSPM approach with static PCA and various detrending methods is then demonstrated on short-term UF case studies in Sections 3.1 and 3.2. We then compare and discuss the long-term performance of MSPM with AD-PCA and univariate adaptive Shewhart fault detection methods in Section 3.3, and we finish with major takeaways and discussion in Section 3.4.

## 2 Methods

Many different univariate and multivariate fault detection methods exist. Univariate methods excel in situations with few variables to monitor but are not ideal for monitoring many variables at once. Furthermore, univariate methods are unable to detect changes in relationships between process variables, which may be of interest. For example, the relationship between membrane permeability and filtrate turbidity should be constant during normal operating conditions once the effects of other water quality parameters have been taken into account. However, if severe fouling takes place or a breakthrough event occurs, this relationship changes. The initial changes to permeability and filtrate turbidity themselves may be small and within operator-set control limits, but the change in the relationship between the two variables may be detected by a PCA-based approach.

Prior to introducing specific fault detection methods, Section 2.1 describes the data, goals of the analysis, and division of variables into monitoring and explanatory groups. Then, a brief description of univariate fault detection methods with Shewhart charts is given in Section 2.2, and multivariate fault detection with PCA is described in Section 2.3. Data and code to replicate the results were reported by Grimm et al.^43^ All computation was performed in the programming language R(44) (version 4.2.1) on a 2021 MacBook Pro.

### 2.1 Pure Water Demonstration Facility

The Pure Water Demonstration facility (PWDF) is located in Calabasas California and is operated by the Las Virgenes-Triunfo Joint Powers Authority. PWDF receives tertiary treated wastewater from the Tapia Water Reclamation Facility (WRF), which typically nitrifies fully, producing an effluent with no detectable ammonia. PWDF further treats the water using UF, reverse osmosis (RO), and an ultraviolet-advanced oxidation process. To mitigate biofouling of membrane systems at PWDF, ammonium sulfate and sodium hypochlorite are added at a target ratio of approximately 4 mg/L CL_2_:1 mg/L NH_3_ to form chloramines. The goal is to maintain a measurable free ammonia residual of 0.5 mg/L NH_3_ to ensure there is no free chlorine present that may damage the RO membranes. Data are generated by instrumentation measuring feed and treated water quality, and 15 min averages are used for this work. For the case studies in Sections 3.1 and 3.2, two short-term time periods are selected. The training periods are selected in order to have a multiday, fault-free training period followed immediately by at least one event determined to be a fault in at least one of the monitoring variables, such as an upward shift in filtrate ammonia. The training periods selected are April 2, 2021–April 13, 2021 (848 observations) and April 27, 2022–May 11, 2022 (1052 observations), and models are tested on days immediately following each training period. History and specifics of the PWDF operation are available in performance reports.^45^ In this work, we focus on monitoring the UF.

The UF process at PWDF is a pilot-scale system for the purposes of gathering performance data to support design and sizing of full-scale operations. To that end, the system is designed to accommodate three different full-scale low-pressure membrane modules, which can each be independently monitored and controlled. Each membrane product is typically operated at a constant flux of 40 gallons per square foot per day (gfd). However, some peak flux testing occurred in 2022 up to 55 gfd. Regardless of flux, the UF membranes were controlled in a dead-end filtration mode, with the filtration production volume set relative to backwash losses to achieve a target recovery of 95%. The specifics of each membrane product installed during the study period are summarized in Table S1 in Supporting Information, and the positions of monitoring equipment are indicated on a process flow diagram in Figure S1.

A separate fault detection model is built for each of the three UF modules, allowing for unit-specific fault detection. Before constructing each fault detection model, we first identify the process monitoring goals. Once the goals are clearly defined, variables that are best associated with those goals are selected as monitoring variables. When the monitoring goals are not directly measured by one of the process variables, a reasonable surrogate can be chosen to represent the goal of interest. For this facility, the three primary monitoring goals are (i) detecting changes in filtrate quality; (ii) detecting changes in upstream water quality; and (iii) membrane fouling. Based on the available data, the monitoring variables that correspond to these goals are filtrate turbidity, filtrate ammonia, and temperature-corrected permeability, respectively. It is important to note that a monitoring variable does not necessarily need to be a variable that the UF process affects. For example, even though ammonia is not removed through UF membranes, it is included as a monitoring variable because unexpected changes in ammonia can indicate upstream water quality changes. This monitoring goal is important to operations because unexpected changes in influent water quality may adversely impact downstream processes (e.g., RO) and put the overall facility goals at risk.

Any variables in the process that are not selected as monitoring variables are used as explanatory variables for detrending. This will remove the variability in the monitoring variables associated with changes in the explanatory variables and is described further in Section 2.3.1. Examples of explanatory variables are pH, flux, and feed turbidity. All explanatory and monitoring variables for this analysis are listed in Table 1.

**Table 1:** List of Explanatory and Monitoring Variables for UF MSPM Models^a^

explanatory variables	monitoring variables
UF feed turbidity	
UF feed temperature	
UF filtrate pH	UF (1, 2, 3) temperature-corrected permeability
UF filtrate ORP	UF (1, 2, 3) filtrate turbidity
UF filtrate total chlorine	UF filtrate ammonia
UF filtrate conductivity	
UF filtrate TOC	
UF backwash flow	
UF (1, 2, 3) flux	

a UF (1, 2, 3) indicates UF-specific measurements for a given variable.

### 2.2 Shewhart Control Chart

Shewhart control charts compare the value of each monitoring variable at a given point in time to its IC mean and raise an alarm if the value exceeds an upper or lower threshold value. The threshold is calculated as a predetermined number of standard deviations away from the monitoring variable’s IC mean or outside a specified confidence interval, both of which are based on the assumption of normality of the monitoring variable. In this work, an adaptive Shewhart chart is used to address nonstationarity by implementing a rolling window of time to recalculate the mean and standard deviation after each day of IC observations. A 99.5% confidence interval, which is approximately 2.8 standard deviations from the mean, is used to determine the Shewhart control limit(s). Additionally, alarms are issued only after 5 consecutive threshold exceedances occur in order to minimize the number of false alarms due to measurement error or noise. Furthermore, lower control limits are only placed on variables where extreme low values are of concern. For example, low UF filtrate turbidity values are not of concern and would not be assigned a lower threshold, but extremely low temperature-corrected permeability values are problematic, so a lower threshold would be computed. This Shewhart control chart is more advanced than is typically used in practice, where control charts typically include only fixed thresholds for both lower and upper limits, even if extreme values in one direction are not practically meaningful.

### 2.3 Multivariate Statistical Process Monitoring

**Figure 1:**
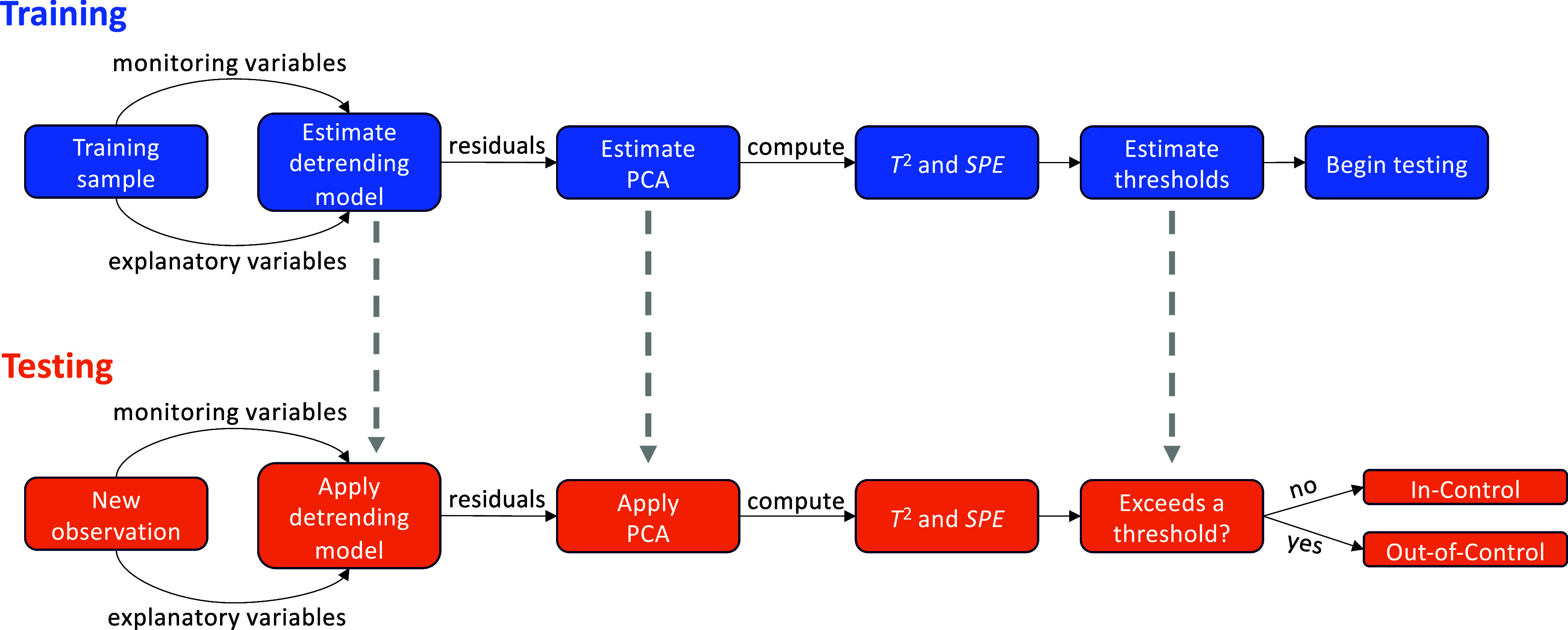
Overall MSPM process steps for training (top) and testing (bottom).

The MSPM approach in this work uses PCA-based methods, which are especially useful when the number of process variables is large. Once the training period, monitoring variables, and explanatory variables are identified, we detrend the monitoring variables by fitting a statistical or ML model. PCA is then applied to the residuals from each detrending model to compute monitoring statistics and their thresholds. When deployed, new observations are detrended, and PCA is applied using the previously fitted models. Then, the monitoring statistics are calculated and compared to the previously defined thresholds. This results in the classification of each new observation as IC or OC (i.e., monitoring statistic is below or above the threshold, respectively). Methods for detrending are described in Section 2.3.1, the MSPM model with AD-PCA is explained in Section 2.3.6, and an overview of the process is given in Figure 1.

#### 2.3.1 Detrending

Before applying MSPM methods, we first detrend the monitoring variables to remove the effect of the explanatory variables. The first step in detrending is to fit a statistical or ML model to observations from the training period. Model predictions are subtracted from the known, observed values to obtain residuals, which represent the variability in the monitoring variable that is not explained by the explanatory variables. We select four regression methods for detrending: adaptive lasso, *k*-nearest neighbors (KNN), random forest (RF), and extreme gradient boosting (XGBoost). The adaptive lasso is computationally fast, interpretable, and simple.^46,47^ KNN is also fast, easy to use, and can fit nonlinear data.^48^ RF and XGBoost tend to produce excellent predictions across many types of data, with XGBoost being newer and more popular recently due to its faster speed and often improved performance compared to RF.^49^Table 2 summarizes important characteristics of each method. In addition to these detrending models, we also consider MSPM without detrending. In the model with no detrending, the observed values of the monitoring variables are used in the MSPM model instead of residuals from a regression model fit.

**Table 2:** Overview of the Characteristics of Each of the Detrending Models Used in This Work

method	nonparametric	speed	linear/nonlinear	# of tuning parameters
adaptive lasso	no	fast	linear	1
KNN	yes	fast	nonlinear	1
RF	yes	slow	nonlinear	>5
XGBoost	yes	slow	nonlinear	>10

In general, some statistical and all ML methods require tuning parameters, often called hyperparameters, that are set by the user and affect model fit. Models with more tuning parameters are more complex and have more flexibility, but can be more difficult to properly fit. Specifically, certain choices of tuning parameters may result in an underfit model that does not learn much from the training data or an overfit model that fits the noise in the training data and does not predict or generalize well to new observations. Therefore, optimal selection of tuning parameters is an essential step when using ML models. Tuning parameters for the methods used in this paper include the penalty parameter λ in adaptive lasso; the value of *k* in KNN, which determines how many neighboring observations to use for model fitting and predictions; the number of decision trees to use in an RF; and the number of boosting iterations for XGBoost.

To determine the best tuning parameters, the training data set is further split into training and validation sets so that different tuning parameters can be tested on withheld data. In this work, a cross-validation (CV) approach is used for tuning parameter selection. In 10-fold CV, the data are divided into 10 equally sized subsamples. A model is trained using nine of the subsamples, and its predictive performance on the remaining subsamples is quantified using root mean squared error (RMSE). The retraining and validation are repeated nine times until each subsample has been assessed, and the overall average RMSE is taken as the CV error. The tuning parameter combination that results in the lowest CV error is selected as the optimal set of model parameters. For the case studies in this paper, we implement 10-fold CV with the train function in the caret package^50^ for all methods except the adaptive lasso. Specific tuning parameters that are varied for each method are reported in Sections 2.3.2, 2.3.3, 2.3.4, and 2.3.5.

#### 2.3.2 Adaptive Lasso

The lasso is an extension of multiple linear regression in which variable selection and coefficient estimation are performed simultaneously.^51^ The adaptive lasso is a modified version of the lasso with additional favorable properties, including consistent variable selection.^46^ This method is very fast, easy to implement, and provides interpretable results. However, it can only model linear relationships between explanatory variables and each monitoring variable.^46,52^ Therefore, even if a very strong nonlinear (e.g., quadratic) relationship exists between an explanatory variable and a monitoring variable, this relationship will not be captured by the adaptive lasso, potentially resulting in a poor model fit and inaccurate model predictions. The value of the tuning parameter, λ, for the adaptive lasso is selected by 10-fold CV, optimizing RMSE, using the cv.glmnet function in the glmnet package.^53^

For observations during the training period, let **y** be the observed values of a monitoring variable; **x**_*j*_ the observed values of the *j*th explanatory variable; β_*j*_ the coefficient for the *j*th explanatory variable; λ a penalty parameter; and  an initial weight for the *j*th coefficient with *j* = 1, 2, ..., *q*. The adaptive lasso coefficient estimates are given by
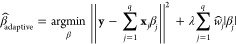
1where the initial weights are taken to be estimates from a ridge regression model.^54^

#### 2.3.3 KNN

In KNN, predictions are made by identifying other observations in the training data that have similar input features. The *k* most similar observations, where *k* is a tuning parameter, are averaged to make the new prediction. In this case, Euclidean distance of the model input feature space is used to measure similarity. Similar to the adaptive lasso, KNN is fast and easy to implement.^55^ Unlike the adaptive lasso, KNN is nonparametric and allows for nonlinear relationships between variables.^56^ However, this model is very simple and can typically be outperformed by other methods. Additionally, this method offers no insight into which explanatory variables are important or how the explanatory variables are related to the monitoring variable. For additional details about KNN, see Hastie et al.^57^

#### 2.3.4 Random Forest

An RF comprises many decision trees that each split a data set into multiple subsets to classify or predict each monitoring variable based on observed values of the explanatory variables. RF works by aggregating results from many decision trees that have been fit to random samples of the data with random subsets of explanatory variables from the original data set.^58^ More specifically, RF splits the data based on levels of explanatory variables and results in final predictions at each terminal node at the end of each “branch” of the decision tree. For a regression problem with a quantitative monitoring variable, the terminal nodes contain the arithmetic mean of the monitoring variable across all training observations associated with that node. Like KNN, RF is able to fit nonlinear relationships in the data. Unlike KNN, RF provides some insight into the importance of explanatory variables to predict the monitoring variable.^49^ Ensemble methods like RF are also able to handle more noise than adaptive lasso and KNN due to the “binning” that takes place at each split. However, one downside of RF is that it is computationally intensive, which can be especially problematic with large data sets. Additional details about RF are given by Hastie et al.^73^ In this paper, the only tuning parameter varied for RF is mtry, which controls the number of variables used by each split within a decision tree. In this work, RF is fit with the randomForest package.^59^

#### 2.3.5 XGBoost

Similar to RF, XGBoost is another method that builds on the decision tree framework.^60^ However, instead of aggregating the results of many different decision trees that have been fit independently, XGBoost iteratively fits a decision tree to improve upon the fit of a previous decision tree, and this “boosting” is repeated many times.^58^ However, boosting requires many more tuning parameters than RF, and XGBoost models are more likely to be overfit.^61^ For application in this paper, we tune the following parameters: eta, the individual tree learning rate; max_depth, the maximum depth of each tree; colsample_bytree, the number of variables used by each tree; subsample, the number of observations supplied to each tree; and nrounds, the number of boosting iterations. In this work, XGBoost is fit with the xgboost package.^62^

#### 2.3.6 Principal Component Analysis

After detrending the monitoring variables, PCA is applied to the residuals, monitoring statistics are computed, and potential faults are then identified. The two monitoring statistics computed after performing PCA are Hotelling’s *T*^2^,^17^ referred to simply as *T*^2^, and squared prediction error (SPE). *T*^2^ measures variability within the PCA model and captures changes in magnitude, while SPE measures variability outside of the PCA model and captures changes in shifts in relationships between variables.^63,64^ In other words, *T*^2^ measures how different each observation is from the others, and SPE measures whether the PCA model is a good fit or not. By using *T*^2^ and SPE together, the information in the set of detrended monitoring variables can be easily plotted, and abnormal behavior is identified if a computed threshold is exceeded at a given point in time.

PCA is performed as follows. Let **r**_*t*_ be the *p* × 1 vector of residuals from the detrending model at time *t* = 1, 2, ..., *n* during the IC training period, where *p* is the number of monitoring variables. The eigenvalue decomposition of the correlation matrix of all the **r**_*t*_’s, **R**, is defined as
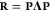
2where **P** is the *p* × *p* projection matrix whose columns are the eigenvectors of **R**, and **Λ** is a diagonal matrix whose diagonal elements are the eigenvalues associated with **P**. PCA is performed by projecting **R** into the principal component subspace by multiplying **R** by the first *k* < *p* columns of **P**, **P**_*k*_, where *k* is the number of principal components required to explain a predetermined percentage of variability in the principal component subspace. This percentage is often selected to be a value between 80 and 99%. Then, *T*^2^ can be computed as
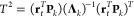
3and SPE^18^ can be computed as
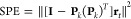
4

where **I** is the identity matrix. Assuming independent observations and multivariate normality of the monitoring variables, the distribution of *T*^2^ is
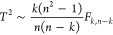
5

The distribution of SPE is approximately
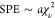
6where *F*_*k*,*n*–*k*_ is an *F*-distribution with *k* and *n* – *k* numerator and denominator degrees of freedom, respectively, and χ_ν_^2^ is a χ^2^-distribution with ν degrees of freedom. The values of *a* and ν are computed by setting the sample mean of the SPE statistics from the training period equal to *a*ν, the sample variance equal to 2*a*^2^ν, and solving for *a* and ν.

Under independence and multivariate normality, the thresholds for classifying new observations as IC or OC are taken as the upper α quantile of each distribution, where α is selected to ensure a desired IC false alarm probability. A value of α = 0.05 corresponds to an expected false alarm rate of one in every 20 observations, which is equivalent to one false alarm every 5 h with a 15 min sampling frequency. Similarly, a value of α = 0.005 yields an average of one false alarm every 50 h.

In practice, the assumptions of independence and multivariate normality are rarely met, especially in complex processes with a high sampling frequency such as water treatment. When observations are not independent or are non-normal, using the theoretical *T*^2^ and SPE thresholds can lead to higher than desired false alarm rates.^65−68^ To avoid issues with parametric thresholds, we use nonparametric thresholds that are computed by finding the 1 – α quantiles of the kernel density estimates of *T*^2^ and SPE during the training period using the Silverman^69^ plug-in bandwidth estimator. The Silverman plug-in bandwidth, *h*, is computed as
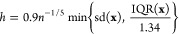
7where **x** represents the set of *T*^2^ or SPE values during the training period, and *n* is the number of observations in the training period.

For the short-term case studies in Section 3, we use static PCA. For the long-term case study in Section 3.3, we incorporate the adaptive and dynamic PCA adjustments by using a moving window to update the model and by including lags of the monitoring variable residuals from detrending in the PCA model. Various methods exist for lag selection,^19,29,70^ and we use the method employed by Klanderman et al.^41^ wherein the residual matrix is augmented with the lag of each variable that has the highest partial autocorrelation function value based on the training period. By augmenting the residual matrix with a single lag for each variable, the number of columns of the residual matrix, **R**, is doubled prior to applying PCA. This method for lag selection is an automatic and computationally efficient approach to lag selection that does not require any iterative selection or simulation. When applying this method to long-term data, we implement an adaptive component to the PCA model by updating the *T*^2^ and SPE thresholds using a moving window approach. After every κ IC observations, the detrending model is refit using the most recent κ IC observations in the training window and excluding the oldest κ observations. In this work, κ = 96, which equals 1 day’s worth of observations. The tuning parameters for the detrending models are reselected through 10-fold CV. Then, we retrain the PCA model, as shown in Figure 1, by re-estimating **R**, **P**, **Λ**, and the nonparametric thresholds for *T*^2^ and SPE.

In the long-term case study in Section 3.3, three tuning parameters become critical for the AD-PCA model. The rejection threshold (α) controls the number of false positives, which should be low. We test two values, α = 0.005 and α = 0.05. The rolling training window size affects the ability of the model to adapt to local conditions. The shorter the training window, the more volatile and reactive the model is to changes in the monitoring variables. We test window sizes of 2 and 12 days. Finally, a single value of the monitoring statistic above the threshold may occur by random chance. Thus, the number of consecutive exceedances required before issuing an alarm can be chosen, and we test either one or five exceedances in a row.

## 3 Results and Discussion

MSPM with static PCA using each detrending method described in Section 2.3.1 is applied to two short-term case studies from PWDF. Results are similar across each UF, so only the results for UF 1 are presented here.

### 3.1 Case Study 1: April 2, 2021–April 23, 2021

**Figure 2:**
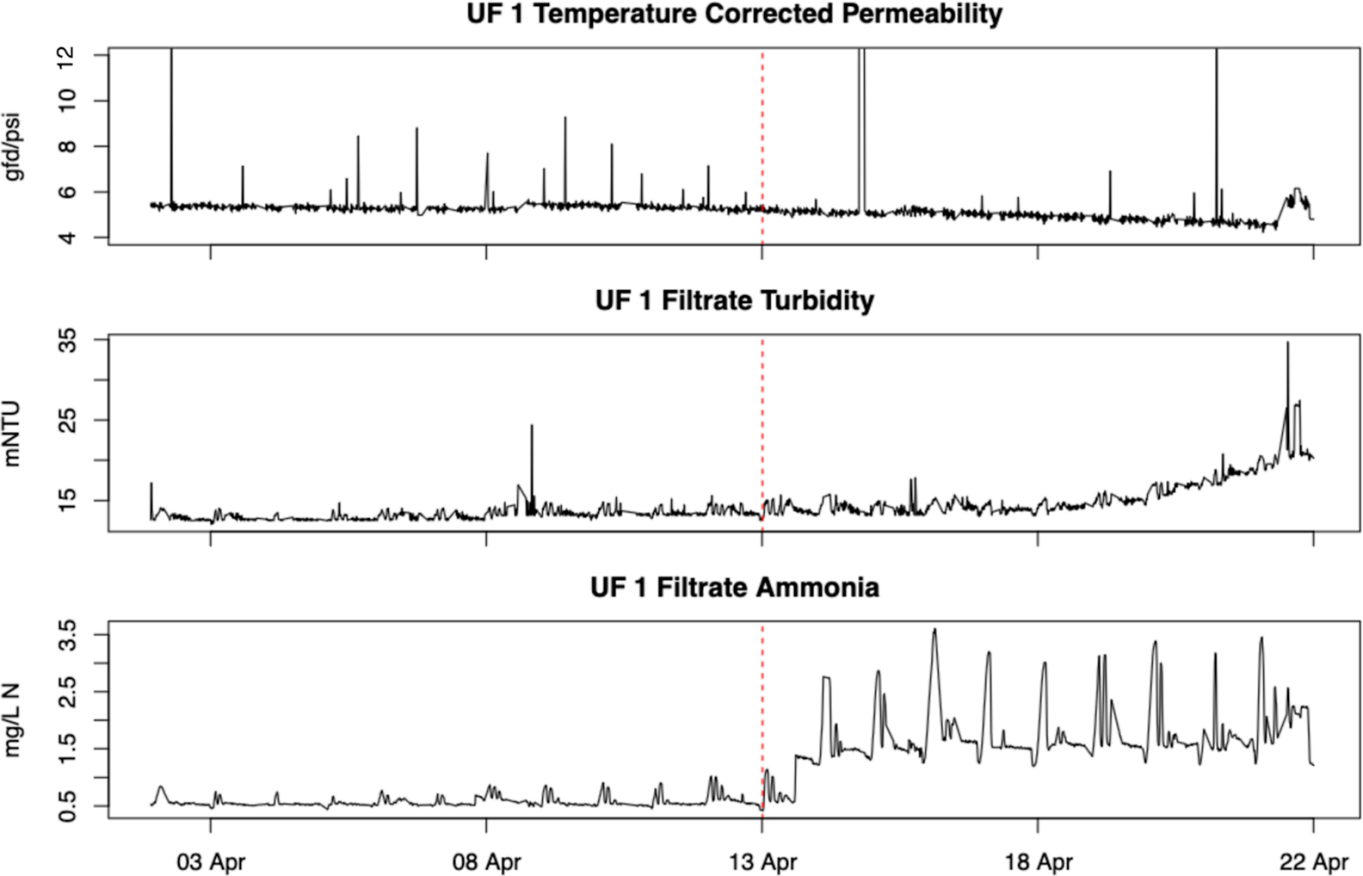
UF 1 monitoring variables plotted over time. The vertical dashed red lines separate the training period (left) from the first several days of testing (right).

Time series plots of each of the UF 1 monitoring variables are given in Figure 2. After the training period, there is a clear upward shift in the filtrate ammonia and a slow upward drift of the filtrate turbidity, but the temperature-corrected permeability is relatively stable with a possible slow drift downward. The increase in ammonia, although within permit limits, was due to an interruption in nitrification at the Tapia WRF. Additionally, the diurnal spikes in ammonia are related to treated effluent pumping cycles to a reclaimed water reservoir located in between Tapia and the PWDF. The subsequent gradual drift in turbidity was later confirmed to be biofouling within the meter measurement cell that was unrelated to membrane integrity. It is suspected that as a consequence of a reduction in nitrification efficiency, there may have been other nutrients that accelerated biofouling potential of the treated effluent, resulting in biofouling of the meter. The conclusion regarding biofouling of the turbidity meter was supported by cleaning of the meter internals, which returned the turbidity meter output to typical baseline levels.

**Figure 3:**
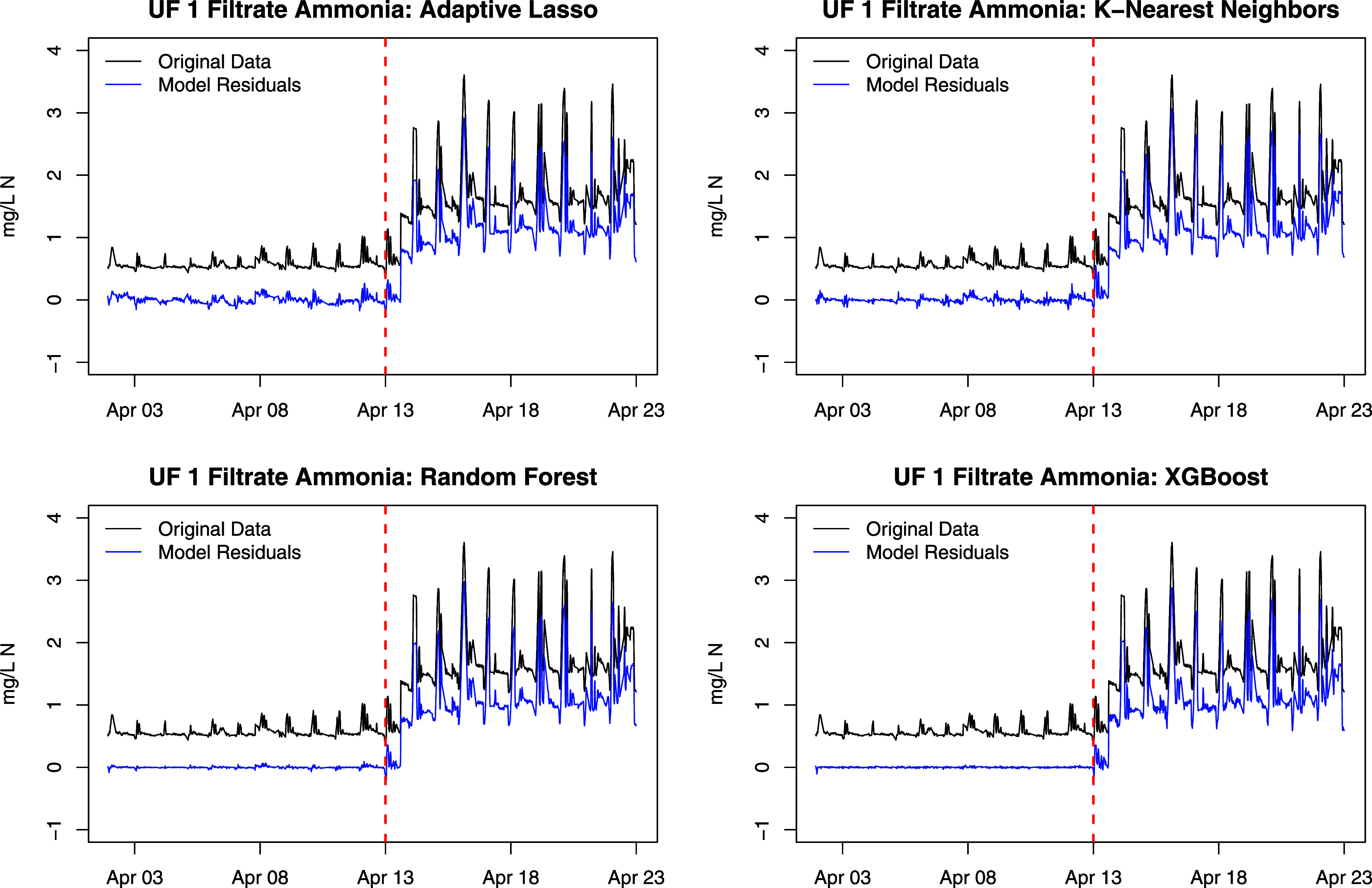
UF 1 filtrate ammonia (black) and residuals from each of four detrending models (blue). Training observations are on the left of the vertical dashed line, and testing observations are on the right.

Each of the detrending methods described in Section 2.3.1 is applied to each monitoring variable to obtain residuals. Plots of the filtrate ammonia and residuals from each detrending method are given in Figure 3. The models produce similar residuals; however, the RF and XGBoost models produce residuals in the training period with very little variability, potentially indicating an overfit model. This overfitting may be addressed by using more training data and performing more extensive model tuning, but uninterrupted periods of time with IC observations are limited, and the tuning process is often time-consuming and therefore difficult to implement in real-time. After detrending, the positive shifts and spikes in ammonia are still present, which indicates that the variation is not expected given the explanatory variables.

**Figure 4:**
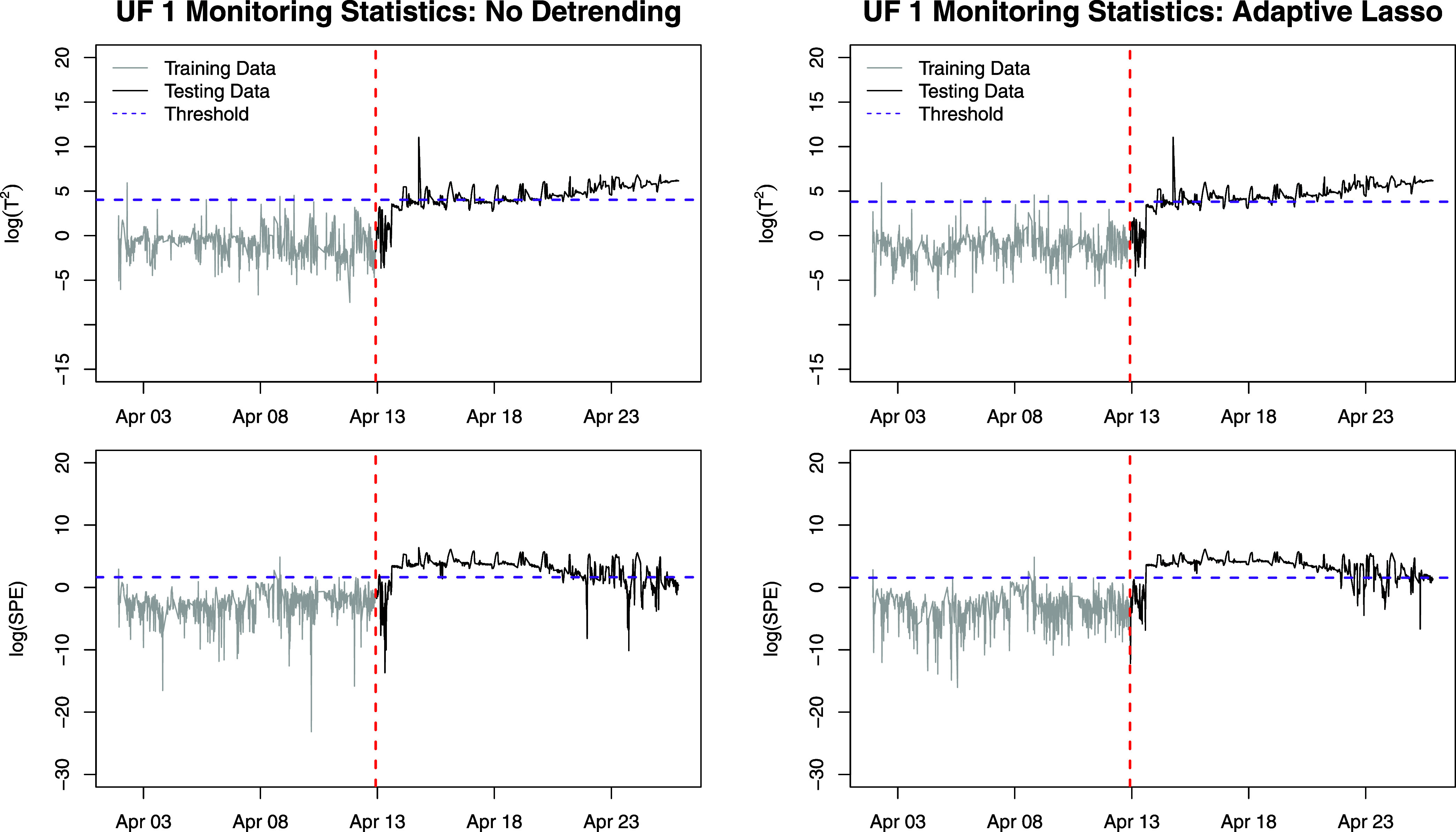
UF 1 monitoring statistic plots with no (left) and adaptive lasso (right) detrending.

After detrending the monitoring variables, we apply PCA, compute *T*^2^ and SPE, and determine the monitoring threshold based on the *T*^2^ and SPE values from the training period. Then, the observations in the testing period can be monitored. Figure 4 gives plots of *T*^2^ and SPE with either no detrending or with adaptive lasso detrending. The model with no detrending has *T*^2^ values that fluctuate around the threshold during the testing period, while the model with adaptive lasso detrending produces *T*^2^ values that exceed the threshold over time faster and more consistently than without detrending. Primary differences in the *T*^2^ exceedances for these two models occur between 16 April and 19 April. During this period, 90.7 and 38.9% of *T*^2^ values exceed their thresholds for the adaptive lasso and no detrending models, respectively (see also Figure S2). The SPE statistics based on these two detrending models are also similar in that the values exceed the threshold, remain above the threshold, and then slowly decrease.

The initial shifts in *T*^2^ and SPE are most likely due to the sudden upward shift in filtrate ammonia shown in Figure 2, and the gradual increase in *T*^2^ values over time is likely due to the upward drift in filtrate turbidity. However, faults are flagged collectively by the MSPM model across all variables instead of individually for each variable. As a result, it is difficult to identify the source or cause of faults that have been detected without using an additional fault isolation method, which is a class of methods used to identify variables that contribute most to the faults that have been detected (see, e.g., Klanderman et al.^41^ and Harrou et al.^71^). Fault isolation is particularly useful when the number of monitoring variables is large, and smaller or multivariate changes are more difficult to diagnose visually.

The monitoring statistic plots based on RF and XGBoost detrending are given in Figure S3. Both of these methods result in *T*^2^ and SPE statistics that quickly exceed the threshold and remain above the threshold. It is important to note, however, that overfit models will result in larger *T*^2^ and SPE values during the testing period even if no shift in any of the variables occurs. These larger values are due to failure of the model to adequately predict the values of the monitoring variables during the testing period. Therefore, the fact that the models with RF or XGBoost detrending produce monitoring statistics that are high above the threshold can be more reflective of the model being overfit than the actual ability of the model to detect faults. As a consequence, overfit models often produce a higher proportion of false alarms than properly fit models.

In summary, all of the detrending methods are able to detect the abnormal behavior in UF 1. Even without detrending, the abnormal behavior is still detected. However, the model without detrending does not detect the fault as definitively as the other methods. Although not shown, the KNN model performs similarly to the no detrending and adaptive lasso models. Due to the overfitting of the RF and XGBoost models and their additional required tuning, the best overall model is the adaptive lasso because it is easy to fit and interpret, and it has good fault detection performance.

### 3.2 Case Study 2: April 27, 2022–May 21, 2022

**Figure 5:**
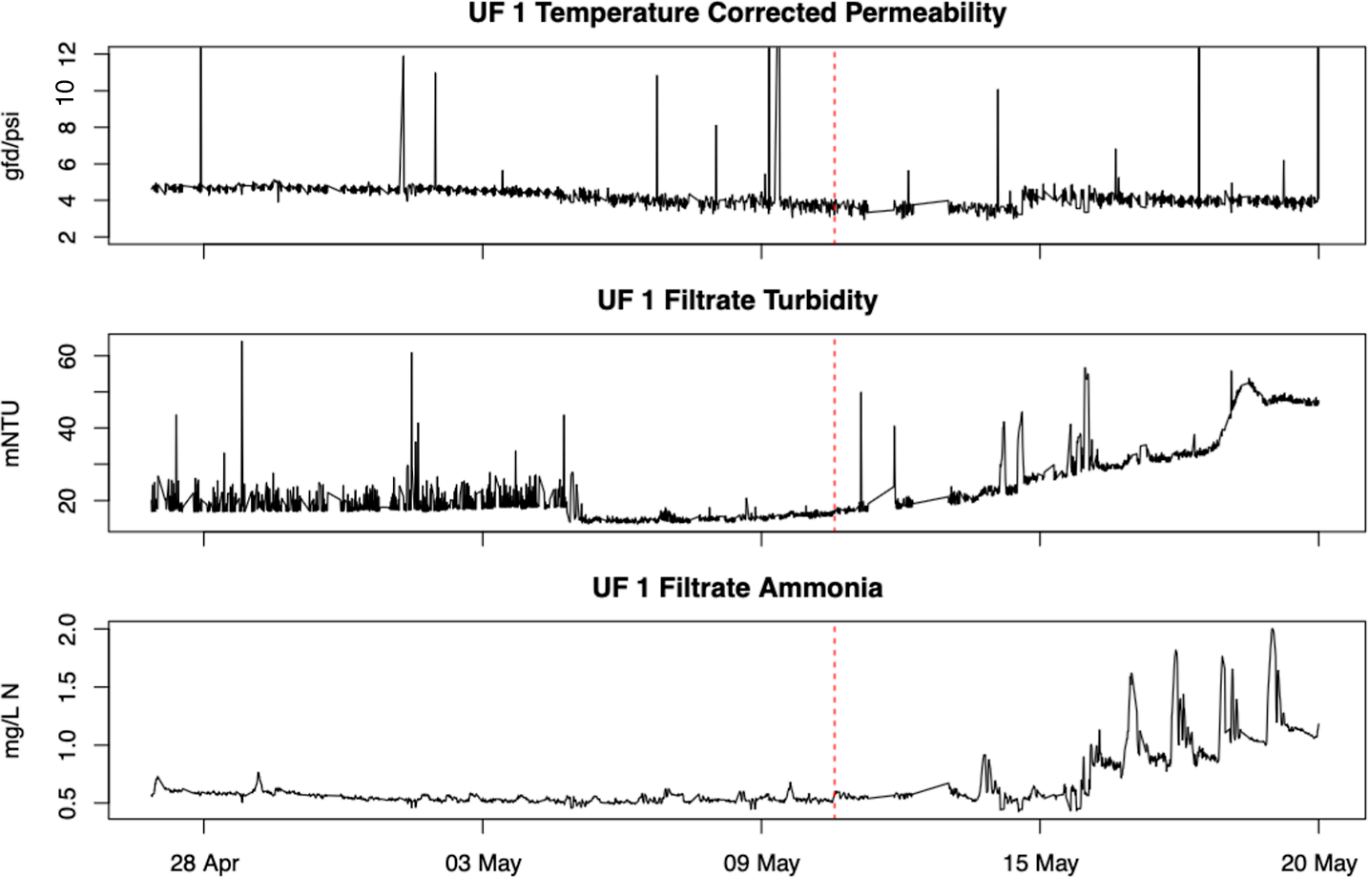
UF 1 monitoring variables plotted over time. The vertical dashed red lines separate the training period (left) from the first several days of testing (right).

**Figure 6:**
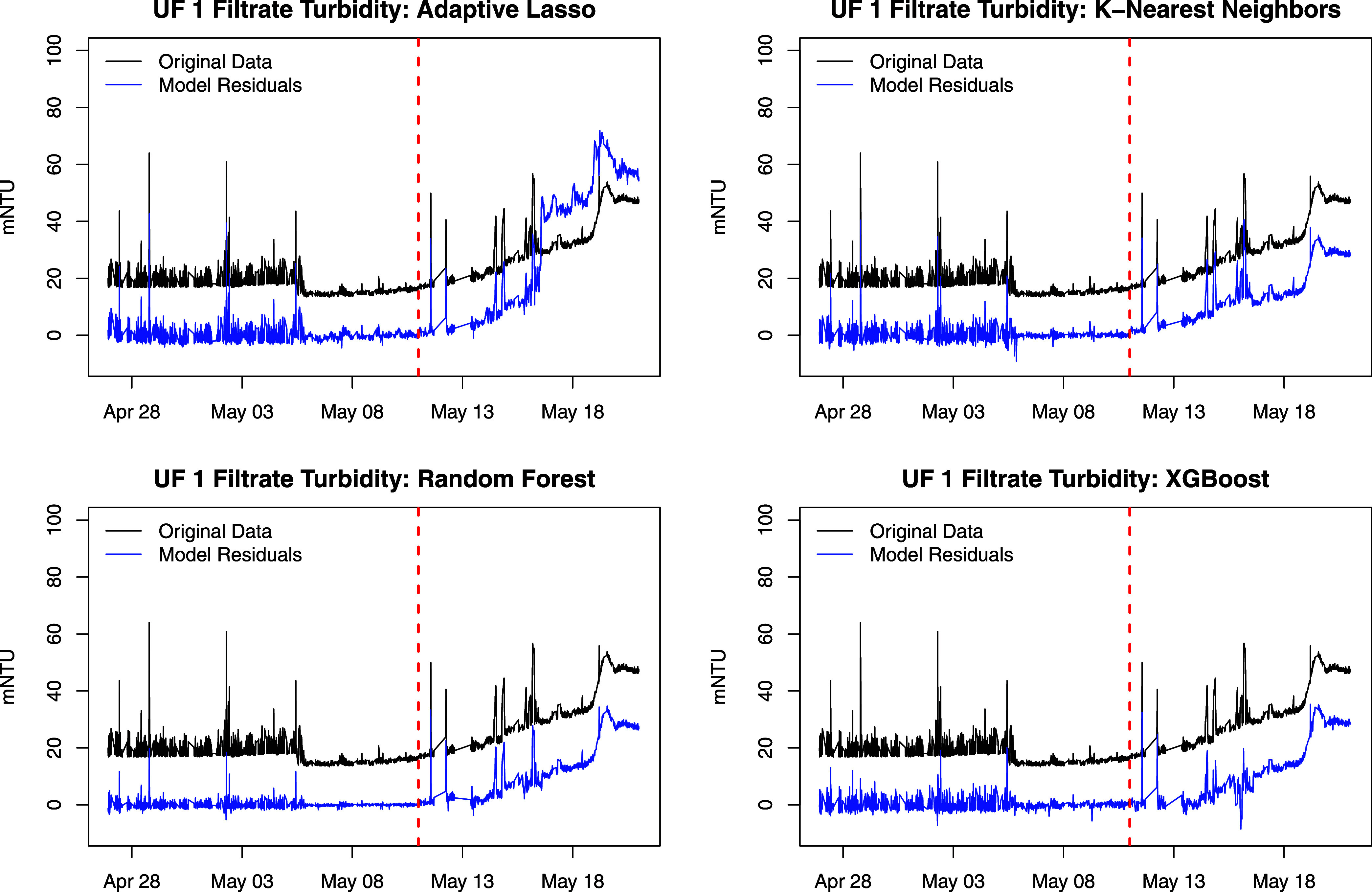
UF 1 filtrate turbidity (black) and residuals from each of four detrending models (blue). Training observations are on the left of the vertical dashed line, and testing observations are on the right.

Time series plots of the UF 1 monitoring variables for the second case study are given in Figure 5. The main faults of interest during the testing period are the upward drift in filtrate turbidity and the increase and spikes in the filtrate ammonia. The temperature-corrected permeability has some brief spikes but generally remains stable over time. Detrending plots for the filtrate turbidity are shown in Figure 6. All of the detrending methods fail to capture the upward trend in turbidity during the testing period, and the adaptive lasso begins to severely underpredict the filtrate turbidity values shortly before May 18.

The *T*^2^ and SPE values for UF 1 based on no detrending and adaptive lasso detrending are given in Figure S4. Both are able to detect the fault with *T*^2^, but differ with SPE. The SPE for the model without detrending has values that fluctuate above and below the threshold, while the SPE for the model with adaptive lasso detrending generally remains above the threshold after detecting the fault. By producing SPE values that consistently exceed the threshold, detrending provides stronger evidence for the presence of the fault. A discussion of the monitoring statistic plots for RF and XGBoost detrending (see Figure S5) is given in the Supporting Information.

### 3.3 Long-Term Case Study

The short-term case studies in this paper only include a few weeks of UF data; each one has several days of IC observations for model training followed shortly afterward by a fault during the testing period. While these case studies demonstrate the importance of detrending and the ability of the MSPM model to detect faults over short periods of time, there is no evidence that the models are able to adequately monitor the UF process over a long period of time. Therefore, it is important to also supplement the short-term case studies with a long-term evaluation of the model performance.

To evaluate long-term fault detection performance, we monitor a period of time that contains known IC and known OC periods. The first IC period is April 2–13, 2021, which is the training period for Case Study 1, and the second IC time period identified is April 27–May 11, 2022, which is the training period for Case Study 2. Known faults occur shortly after each of these training periods. Ideally, the model should be able to identify any observations or periods of time with abnormal behavior during the 379 days between the two training periods (from April 13, 2021 to April 27, 2022), and by the time of the second known IC period, observations should be classified as IC. Then, during the period between May 12 and May 25 when a known fault occurs, observations should be classified as OC and raise an alarm. Because of the speed, ease of use, and performance of the model with adaptive lasso detrending in the short term case studies, the AD-PCA model with adaptive lasso detrending is fit to the UF 1 data from April 2, 2021 through May 25, 2022. As a benchmark, an adaptive Shewhart control chart is fit to each monitoring variable during the same time period. Plots of the three monitoring variables during this long time period are included in Figure 9 in Section 3.3.2.

#### 3.3.1 MSPM with AD-PCA

**Figure 7:**
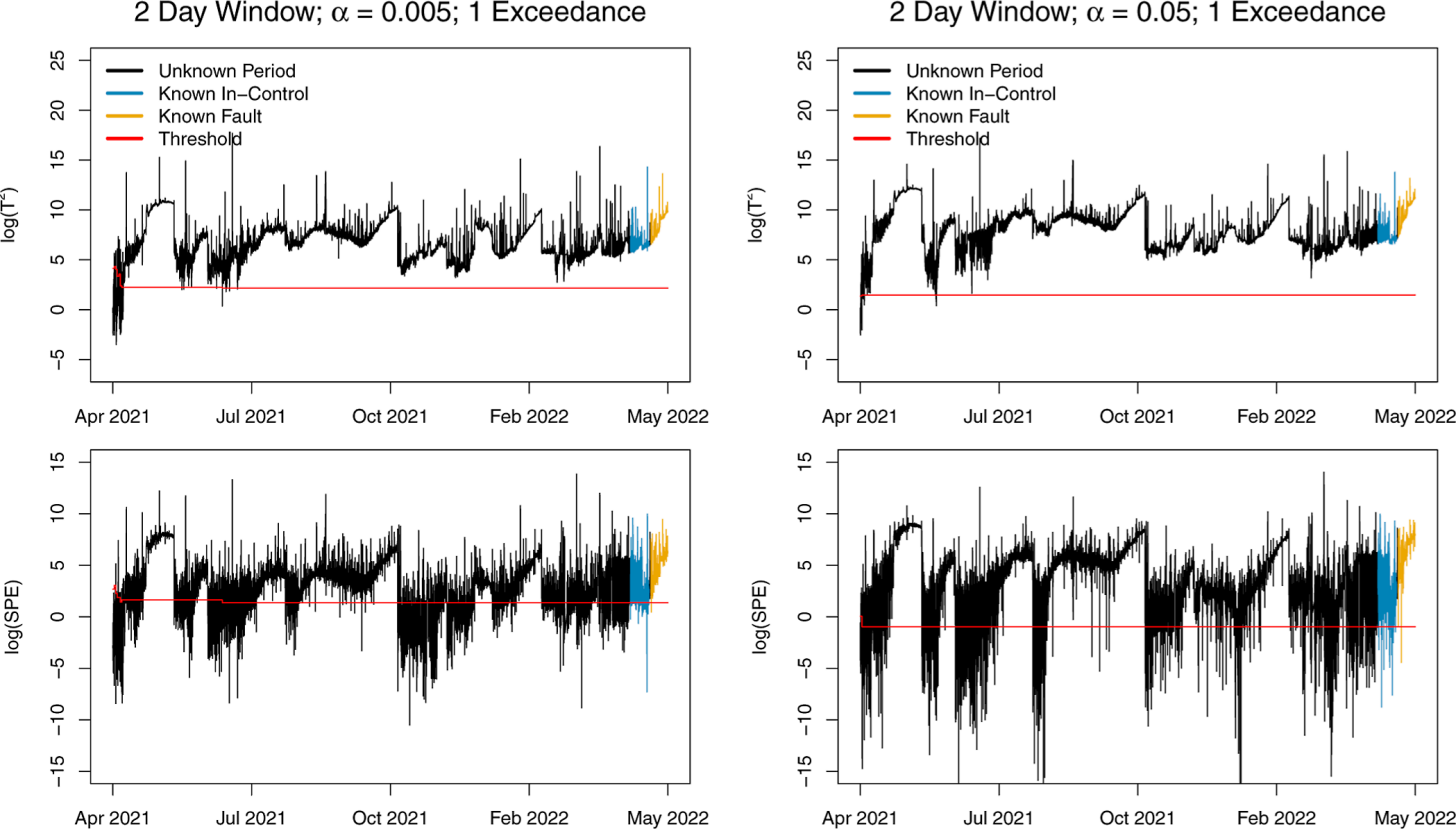
*T*^2^ and SPE with 2 day training window, updating after every 1 day of IC observations using a rejection threshold of α = 0.005 (left) and α = 0.05 (right).

Figures 7 and S7 show results for both values of α and with one threshold exceedance needed to issue an alarm for either a 2 day window or 12 day window, respectively. For each of these model fits, the model is updated after every 96 IC observations (1 day), where IC observations are defined as only the observations where both *T*^2^ and SPE are below the threshold. In Figures 7 and S7, long periods of time are classified by the model to be OC. As a result, the model fails to update, as indicated by the unchanging threshold, and consequently classifies entire months as OC. When the monitoring statistic is colored blue, this represents a period of time where the process is known to be IC and should not exceed the red threshold. The yellow monitoring statistic indicates a period when a fault is known to occur and should be above the threshold. In both of these figures, the model rarely updates, and almost all observations are above the threshold, indicating that the model is unable to correctly identify the known IC and known fault periods at the end of the time period.

Even though MSPM with PCA is shown to perform well on the short-term case studies when a proper IC training period can be identified prior to testing, the long-term performance is not acceptable. One solution could be to manually update the model after a fault has been resolved. However, this approach requires manual intervention and is less suitable for automatic real-time application. To address this, we test requiring multiple, sequential observations to be above the *T*^2^ or SPE thresholds before issuing an alarm.

**Figure 8:**
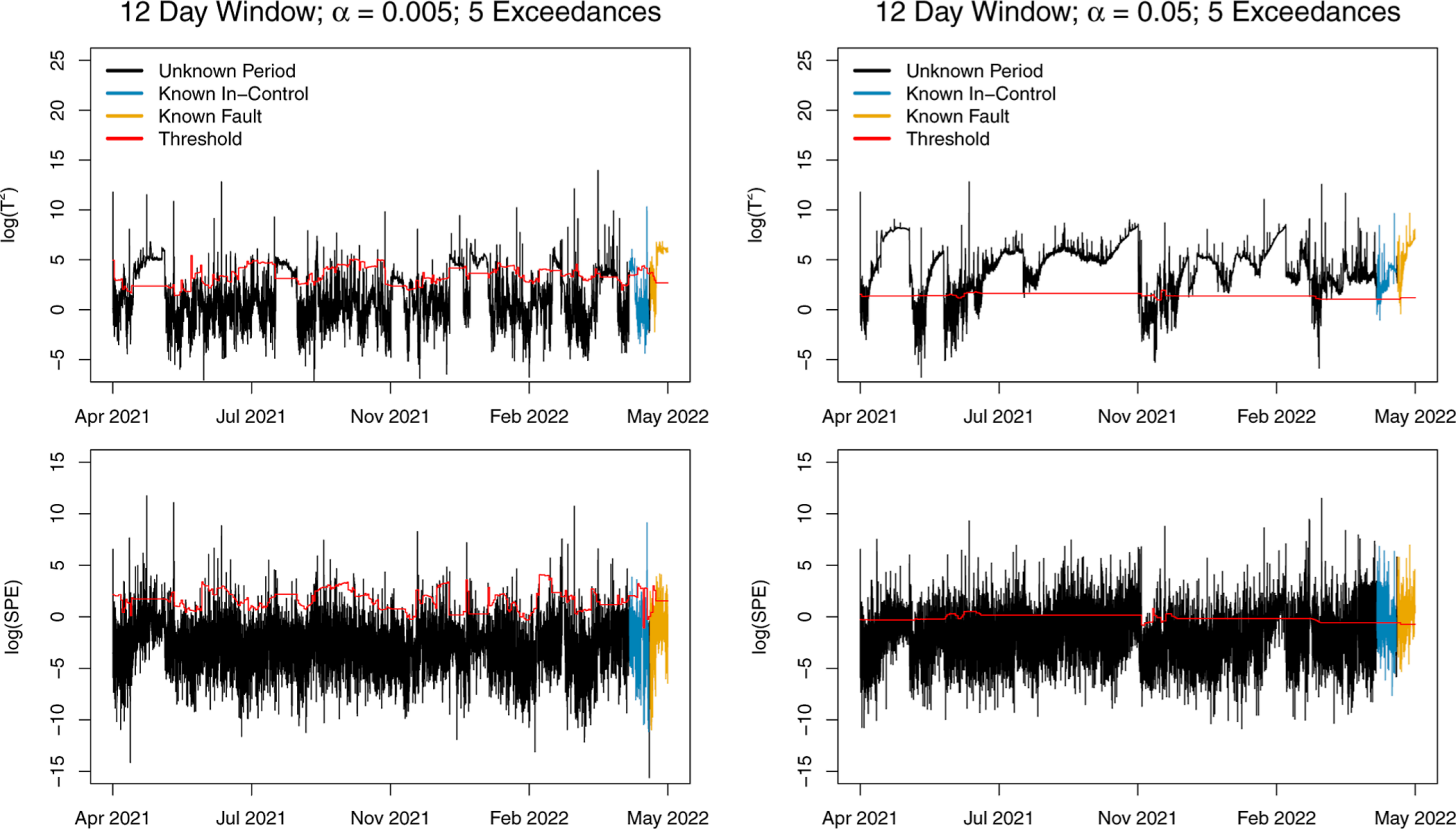
*T*^2^ and SPE with 12 day training window, updating after every 1 day of IC observations using a rejection threshold of α = 0.005 (left) or α = 0.05 (right). Alarms are only issued after 5 or more consecutive threshold exceedances in either *T*^2^ or SPE.

Figure 8 shows results from the AD-PCA model when applied to the same time period, using a 12 day training window, updating after every 1 day of IC observations, with α = 0.005 or α = 0.05. In this figure, OC observations are defined as those such that there are 5 or more consecutive threshold exceedances between either *T*^2^ or SPE. The results when α = 0.005 are more consistent with our expectations of how the model should adapt: several faults are identified during the unknown period, but the model continues to update and adapt, as indicated by the changing threshold. In contrast with 1 exceedance for an observation to be OC, the model with 5 exceedances, α = 0.005, and a 12 day training window does not flag several months in a row as OC. Furthermore, the second IC training period is shown to be mostly classified as IC by the model, and observations are flagged again during the known fault period. However, when α = 0.05, the model still fails to adapt, and observations during the second training period are mostly classified as faults. Plots and a discussion of results for the 2 day training window with 5 consecutive exceedances to issue an alarm are given in Supporting Information.

An “ideal” model would be expected to retrain intermittently throughout the unknown period (where IC and OC conditions are both present but observations are not explicitly labeled as such) and the second training period (known IC). Additionally, the “ideal” model would not flag observations in the second training period and then flag the observations in the second known OC period and stop retraining. Thus, the best performing model in Table 3 would have a low percentage of flagged known IC observations (false alarm rate) and a high percentage of flagged known OC observations (true alarm rate). In Table S2, the model with a training window size of 12 days, α = 0.005, and alarms issued after 5 consecutive exceedances is the only model to retrain during a portion of the second known IC period and has the lowest false alarm rate during the second training period. The only other model that retrains during the known fault period is the model with a 12 day training window, α = 0.05, and 5 consecutive exceedances to issue an alarm. During the known fault period, all models identify the fault with a high percentage of observations exceeding the threshold. All models except one have nearly a 100% alarm rate during the known fault period, which is not surprising due to their near 100% alarm rate during the known IC period and lack of model retraining. However, the model with a 12 day training window, α = 0.005, and 5 consecutive exceedances to issue an alarm is able to distinguish between the known IC and known fault periods and retrain during the known IC period. As a result, this model achieves a lower alarm rate during the IC period and an increased alarm rate during the known fault period. Because no other models retrain during the known IC period and because all of the other models flag almost all known IC observations as OC, the set of tuning parameters that yield the best results are a window size of 12 days, α = 0.005, and 5 consecutive exceedances to issue an alarm.

**Table 3:** Percent of Observations Exceeding the Threshold for Each Window Size, Level of α, and Number of Exceedances in a Row to Trigger an Alarm^a^

window size (days)	α	alarm (# of obs.)	unknown period (*n* = 35,954)		known IC (*n* = 1419)		known fault (*n* = 1238)	
			*T*^2^	SPE	*T*^2^	SPE	*T*^2^	SPE
2	0.005	1	98.1 (36,229)	73.3 (27,067)	100 (1419)	90.5 (1284)	100 (1238)	99.9 (1237)
		5	75.3 (27,813)	56.8 (20,952)	100 (1419)	96.6 (1371)	99.8 (1236)	56.8 (703)
	0.05	1	99.6 (36,776)	92.5 (34,150)	100 (1419)	97.8 (1388)	100 (1238)	99.9 (1237)
		5	96.2 (35,503)	83.0 (30,650)	100 (1419)	96.7 (1372)	100 (1238)	99.0 (1226)
12	0.005	1	80.2 (28,827)	13.5 (4844)	100 (1419)	50.1 (711)	100 (1238)	39.3 (487)
		5	26.8 (9635)	1.4 (507)	25.9 (368)	1.4 (20)	75.8 (939)	7.3 (90)
	0.05	1	96.4 (34,644)	31.2 (11,411)	100 (1419)	73.6 (1044)	100 (1238)	85.7 (1061)
		5	85.0 (30,550)	23.6 (8503)	95.6 (1357)	60.4 (857)	98.5 (1219)	81.7 (1011)

a The number of observations in each period (*n*) is also given, and entries in the table show the number of observations exceeding the threshold in parentheses to the right of the percentage.

The primary drawback of increasing the number of exceedances before raising an alarm is delayed detection of faults. When a fault truly occurs, detection of the fault will be delayed by four observations and be detected on the fifth consecutive observation with a threshold exceedance, which translates to a 1 h delay with a 15 min sampling frequency. Failing to increase the number of exceedances before raising an alarm results in more observations being classified as OC, which could lead to wasted resources or complacency and abandonment of the fault detection system. In spite of this limitation, faults can still be detected much faster than they would be with current membrane monitoring practice, which entails the usage of a daily pressure decay test to evaluate membrane integrity.^72^

#### 3.3.2 Shewhart Control Charts

To compare MSPM with AD-PCA to simpler and more commonly used methods, we apply a Shewhart control chart to each of the three monitoring variables. The control charts are applied over the same long-term time period of UF 1 data from April 2, 2021, through May 25, 2022, with a rolling training window of 12 days, α = 0.005, a 1-day model retraining, and alarms issued after 5 consecutive exceedances.

**Figure 9:**
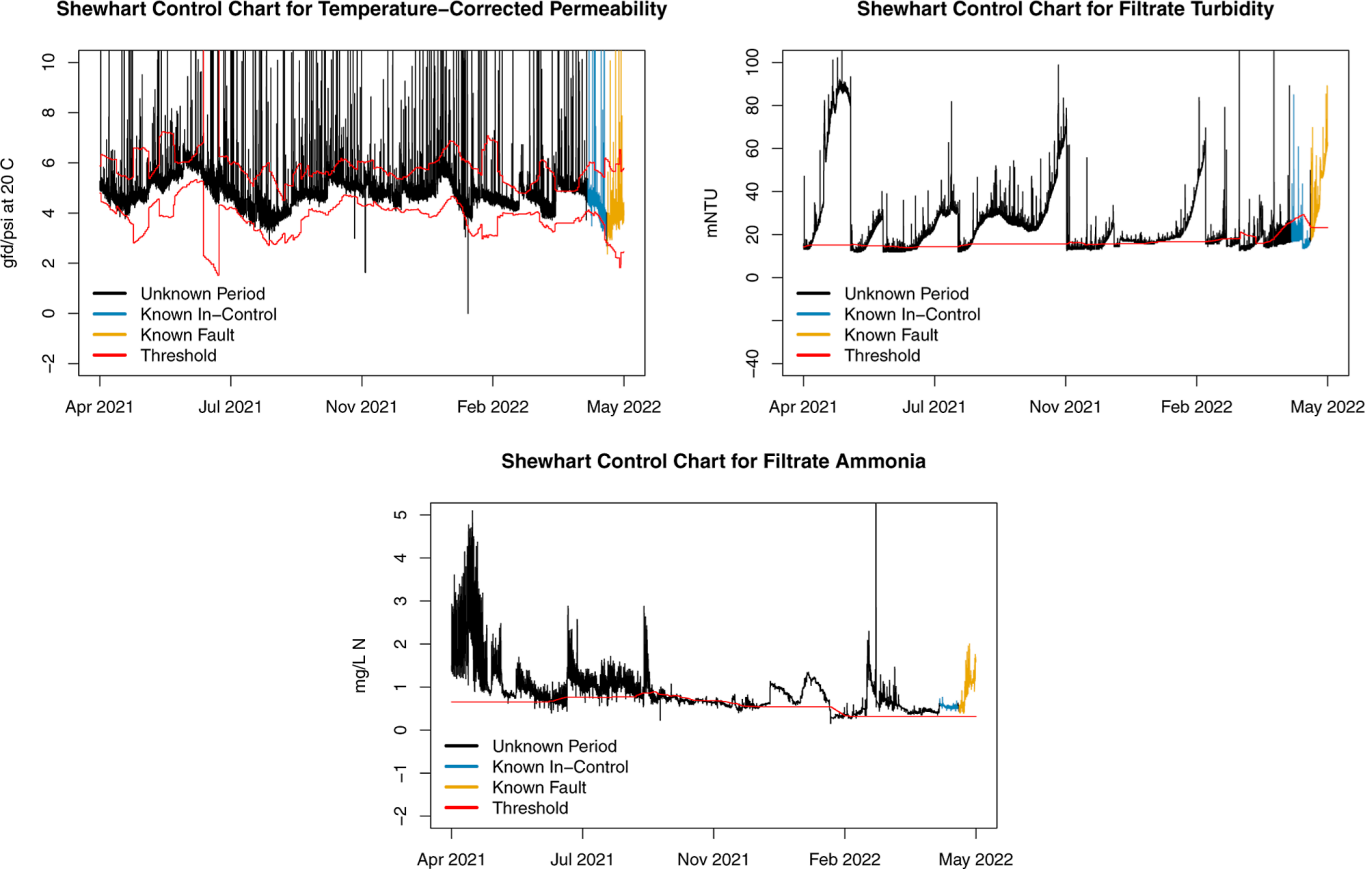
Univariate control charts for each monitoring variable. Thresholds are shown in red, and each time period is shown with a different color.

The univariate control charts for each monitoring variable during the unknown period, known IC, and known fault periods are shown in Figure 9. Additionally, Tables 4 and 5 contain the count and percentage of threshold exceedances and days retrained during each period, respectively. The temperature-corrected permeability values mostly remain within the upper and lower thresholds, there are no obvious faulty periods, and the values remain mostly IC for both the known IC and known fault periods at the end. As a result, approximately 10% of observations exceed the threshold during the testing period, 5% during the known IC period, and 1.5% during the known fault period. Because few observations exceed the thresholds during each period, the control chart for temperature-corrected permeability has the largest percentage of days where the model retrained relative to the other monitoring variables. Based on this control chart, there appear to be no major unexpected changes in temperature-corrected permeability.

**Table 4:** Percent of Observations That Exceed the Threshold for a 12 Day Training Window, α = 0.005, 5 Exceedances in a Row to Trigger an Alarm^a^

variable	unknown period (*n* = 36,014)	known IC (*n* = 1419)	known fault (*n* = 1238)
temp.-corrected permeability	9.8 (3519)	5.2 (74)	1.5 (19)
filtrate turbidity	64.0 (23,051)	1.6 (23)	88.5 (1096)
filtrate ammonia	74.8 (26,952)	100.0 (1419)	100.0 (1238)

a The number of observations in each period (*n*) is also given, and entries in the table show the number of observations that exceed the threshold in parentheses to the right of the percentage.

**Table 5:** Percent of Days Retrained During Each Period for a 12 day Training Window, α = 0.005, and 5 Exceedances in a Row to Trigger an Alarm^a^

variable	unknown period (384)	known IC (14)	known fault (12)
temp.-corrected permeability	94.8 (364)	100 (14)	33.3 (4)
filtrate turbidity	41.4 (159)	50 (7)	0 (0)
filtrate ammonia	27.9 (107)	0 (0)	0 (0)

a The number of days during each period (*d*) is also given, and entries in the table show the number of retrained days in parentheses to the right of the percentage.

Filtrate turbidity has a rapid, sustained increase shortly after the testing period begins, several periods of consistent threshold exceedances occur during the unknown period, and the values occasionally return beneath the threshold before spiking up again. During and shortly before the known IC period, the filtrate turbidity is generally less than the threshold, and the values spike up and exceed the threshold during the known fault period. During the long unknown period, 64% of observations exceed the threshold due to the repeated sustained spikes in turbidity, but only 1.62% of observations are flagged during the known IC period. A large spike in turbidity occurs shortly after the known fault period begins, causing 88.5% of the observations during that period to exceed the threshold.

The upward shift in filtrate ammonia occurs exactly after the end of the initial training period, resulting in immediate threshold exceedances at the beginning of the unknown period. Although the filtrate ammonia values slowly decrease over time, the majority of values (74.8%) during the unknown period exceed the threshold. Furthermore, due to additional large spikes after February 2022 and failure of the model to retrain quickly enough, the entirety of observations during the known IC period exceed the threshold. As a result, once the known fault period begins, the observations move further above the threshold, causing all observations to continue to be flagged as OC.

### 3.4 Discussion

Fault detection research typically focuses on evaluating model performance over a short period of time using hand-picked case studies. This approach allows for rapid testing of new methods, but real-world implementation of fault detection models requires monitoring process variables over long periods of time. We show that MSPM with AD-PCA is effective for the short-term case studies discussed in this work. The adaptive lasso is found to be the best detrending method due to its ease of use and fault detection performance compared to more complex and computationally intensive ML models. Then, tuning parameters for AD-PCA with adaptive lasso detrending over a long time period are evaluated, and the best results are compared to adaptive univariate Shewhart control charts.

In practice, univariate control charts are not as flexible as AD-PCA and would produce less reliable results. For some scenarios, univariate control charts perform adequately, and more complex multivariate methods are not needed. This is true primarily when the number of process variables is small. However, in processes with many monitoring variables, the AD-PCA method becomes increasingly attractive due to its ability to condense information from all monitoring variables into one or two monitoring statistics. Additionally, changes in relationships between monitoring variables may be of particular interest in some processes, in which case MSPM must be used instead of univariate control charts. Overall, the AD-PCA method performs well at full-scale, and careful selection of model tuning parameters will ensure that users realize its full benefits.

Among the AD-PCA models compared for long-term monitoring, the best model uses a 12 day training period with α = 0.005 and 5 consecutive threshold exceedances to trigger an alarm. Univariate control charts of each monitoring variable that use the same training period, value of α, and number of exceedances are also able to identify faults. However, not all of the univariate control charts are reliable; the univariate chart for ammonia flagged periods as OC that should be classified as IC. Additionally, larger proportions of filtrate turbidity and filtrate ammonia values are flagged as OC during the long unknown period compared to *T*^2^ and SPE. Although variable-specific fault diagnosis information is readily available from the univariate charts, these charts appear to result in too many false alarms.

The AD-PCA approach is able to take into account both the information from all of the explanatory variables and the relationships between the monitoring variables, which the univariate control charts cannot do, and contributes to the sensitivity of the method. Although the increase in ammonia during the two short-term case studies appears to be substantial, it is only on the order of 1 mg/L-N from the baseline. This level would not impact permit compliance but is of interest to operations. Contextually, data analysis to the level of granularity conducted in this work is not required for permit compliance. However, when considering implementation of advanced controls to stabilize production efficiency and water quality for potable reuse, the ability to automate identification of low magnitude but important changes in water quality is important. Ideally, full-scale facilities would not need to be staffed around the clock. In such cases, it is important to automate the detection of significant deviation in process inputs, outputs, or behavior, without increasing the frequency of nuisance alarms.

An important consideration when implementing AD-PCA on different data sets is the selection of various tuning parameters, including the false alarm rate (α), the moving training window size, and the number of exceedances before an alarm is issued. Tuning parameter selection should be done separately for each data set because characteristics in the data sets may vary widely. However, tuning only needs to be done once, prior to initial usage of the AD-PCA model, unless substantial changes in the process occur that require further tuning. Potential tuning parameters to explore include values of α between 0.1 and 0.001; window sizes ranging from a few days to a few weeks; between 1 and 10 consecutive exceedances before issuing an alarm. Values of α should be selected to ensure quick detection of faults without causing excessive false alarms. The window sizes and number of consecutive exceedances depend heavily on the nature of the process and sampling frequency of the data. To select tuning parameters, we recommend the approach used in this work: first, identify known IC periods followed by known faults. Then, evaluate model performance across a range of tuning parameter combinations, and select the combination that yields the desired performance of *T*^2^ and SPE.

## Supporting Information Available

Diagram and additional details of the UF process, monitoring statistic plots for all detrending models and case studies, and a complete table of results for the long-term modeling study (PDF)
