# Supplementary material for: Long-Term Statistical Process Monitoring of an Ultrafiltration Water Treatment Process
Source: ACS ES T Eng. 2024 May 31;4(6):1492–506. doi: 10.1021/acsestengg.4c00042 (PMC11184555; doi:10.1021/acsestengg.4c00042)
Supplement: Supplementary file 1 — ee4c00042_si_001.pdf [file ee4c00042_si_001.pdf]

Long-term Statistical Process Monitoring of an Ultrafiltration Water Treatment Process

Taylor R. Grimm,[†] Amos Branch,[‡] Kyle A. Thompson,[‡] Andrew Salveson,[‡] John Zhao,[¶] Darrell Johnson,[¶] Amanda S. Hering,[†] and Kathryn B. Newhart[*,§]

†*Department of Statistical Science, Baylor University, Waco, TX, 76798, USA*

‡*Carollo Engineers, Inc., Walnut Creek, CA, 94598, USA*

¶*Las Virgenes Municipal Water District, Calabasas, CA, 91302, USA*

§*Department of Geography and Environmental Engineering, United States Military Academy, West Point, NY, 10996, USA*

E-mail: kathryn.newhart@westpoint.edu

Table S1: UF products installed in the PWDF during the study period.

	UF1	UF2	UF3
Vendor	DuPont	Pall	Toray
Model	SFD-2880XP	UNA-620A	HFUG-2020AN
Filtration Area (ft^2)	829	538	969
Reported Nominal Pore Size (μm)	0.03	0.10	0.01

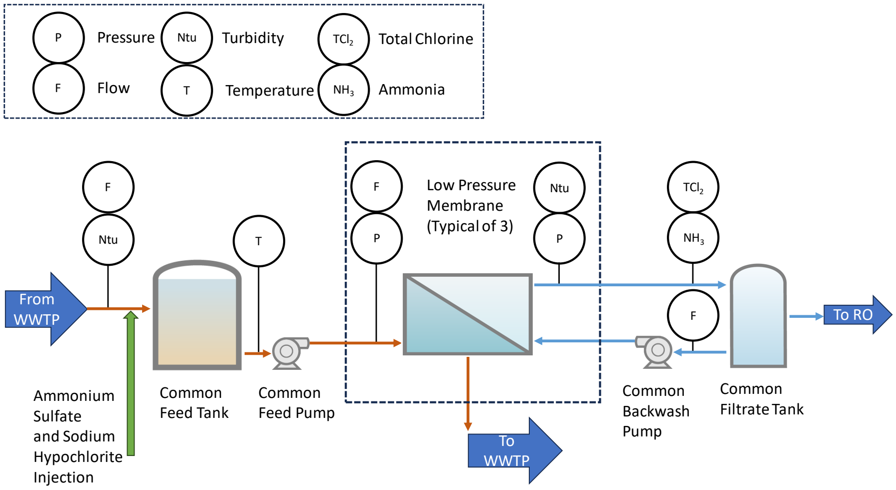

Figure S1: Ultrafiltration process flow diagram indicating positions of monitoring equipment.

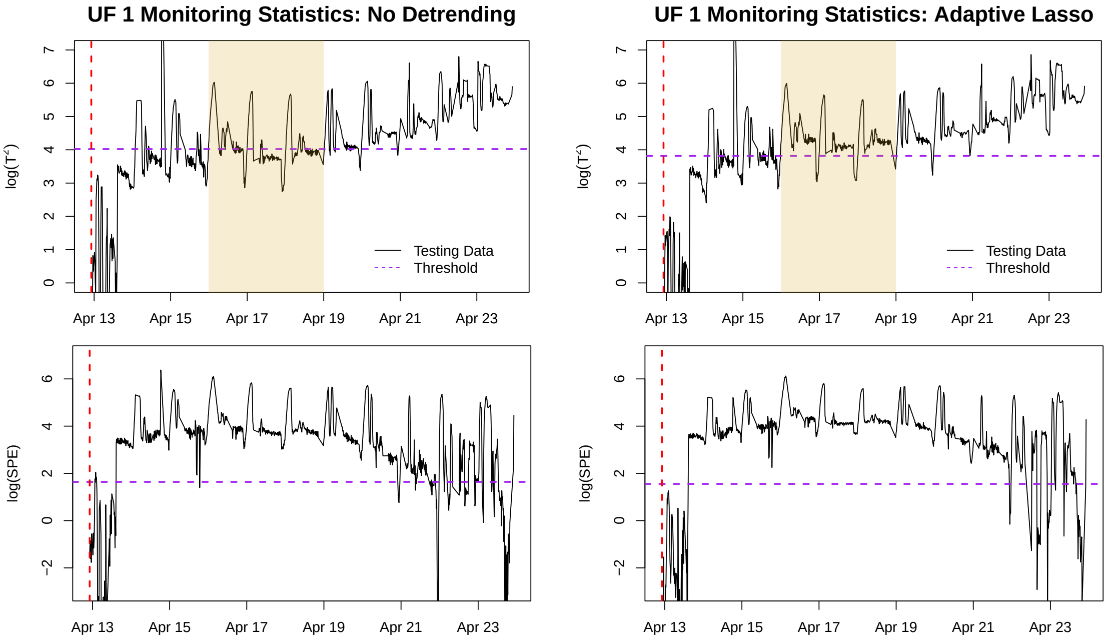

Figure S2: Testing period UF 1 monitoring statistic plots for the 2021 case study with no (left) and adaptive lasso (right) detrending. A region is shaded on the T^2 plots to highlight the differences in T^2 exceedances between the models.

Plots of the monitoring statistics for the two case studies with different detrending methods are given in Figures S3, S4, and S5. For RF and XGBoost detrending in Figures S3 and S5, the T^2 values remain above the threshold after detecting the fault, but XGBoost has more fluctuations above and below the threshold for SPE during the testing period.

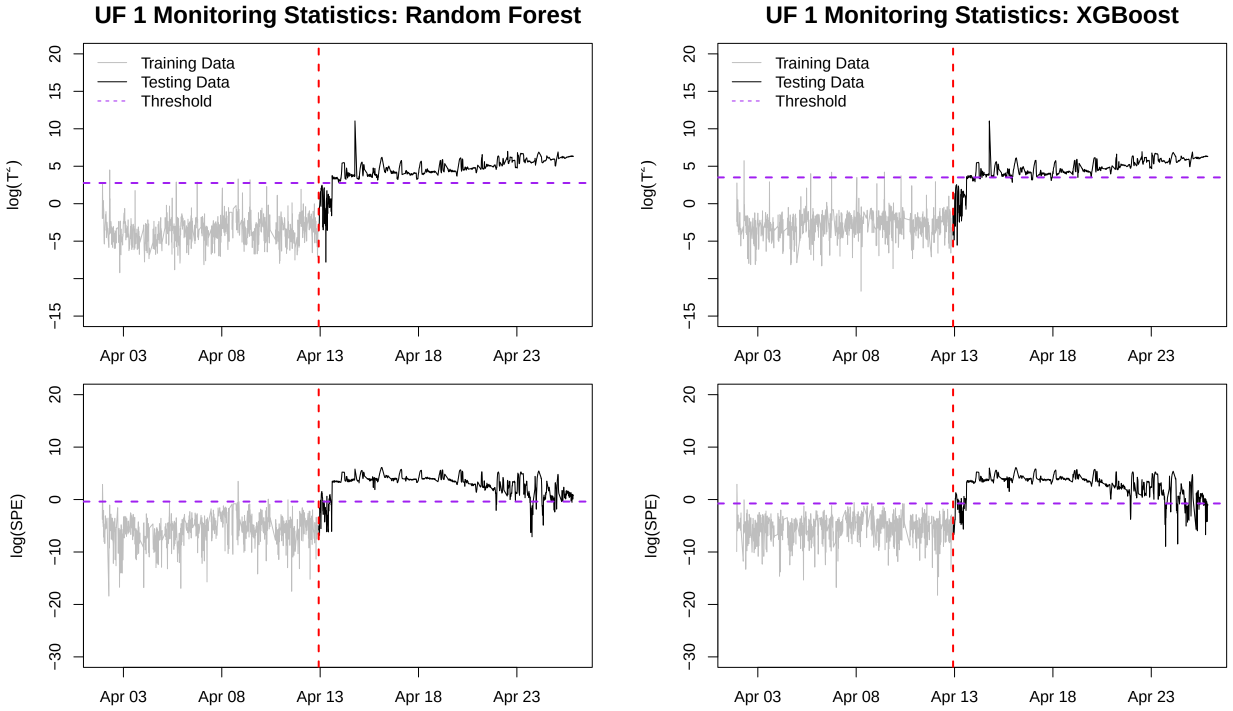

Figure S3: UF 1 monitoring statistic plots in the 2021 case study with RF (left) and XGBoost (right) detrending.

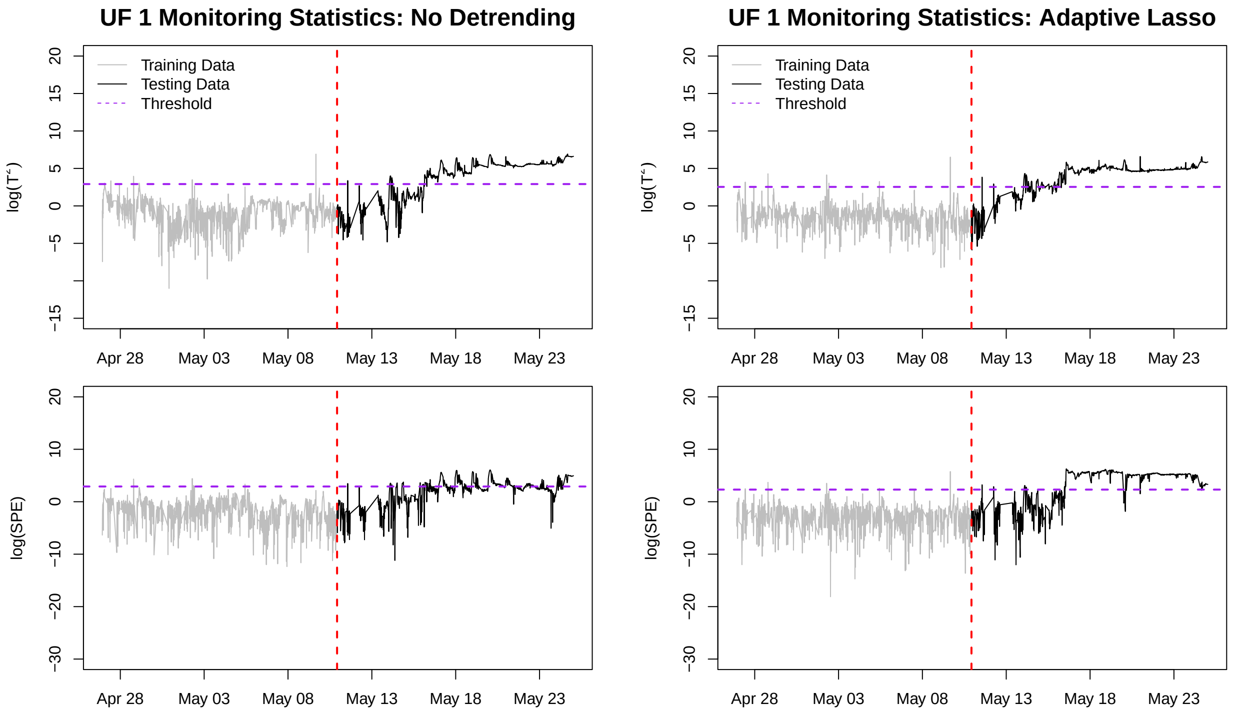

Figure S4: UF 1 monitoring statistic plots in the 2022 case study with no (left) and adaptive lasso (right) detrending.

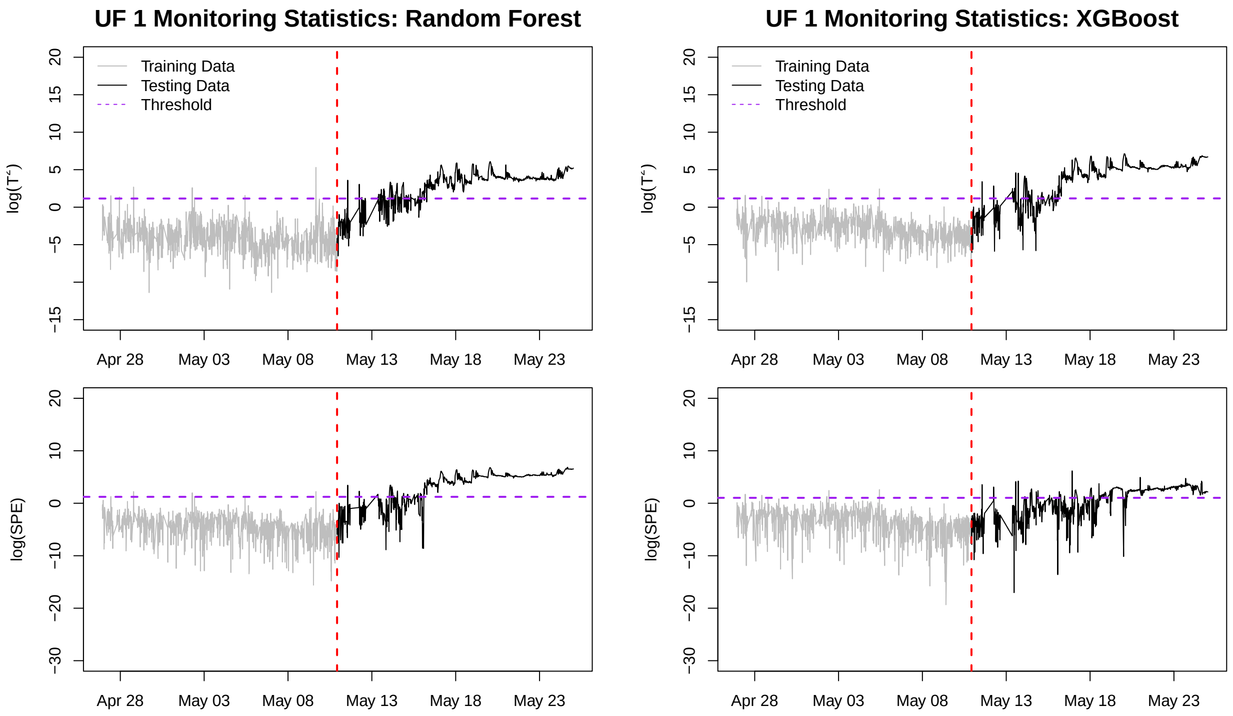

Figure S5: UF 1 monitoring statistic plots in the 2022 case study with RF (left) and XGBoost (right) detrending.

Results from fitting AD-PCA over the long-term time period are shown in Figures S6 and S7. Using 5 exceedances before issuing an alarm results in more retrainings during the unknown period than using just 1 exceedance to issue an alarm, but using a 2 day training window leads to almost all observations being classified as OC after October 2021.

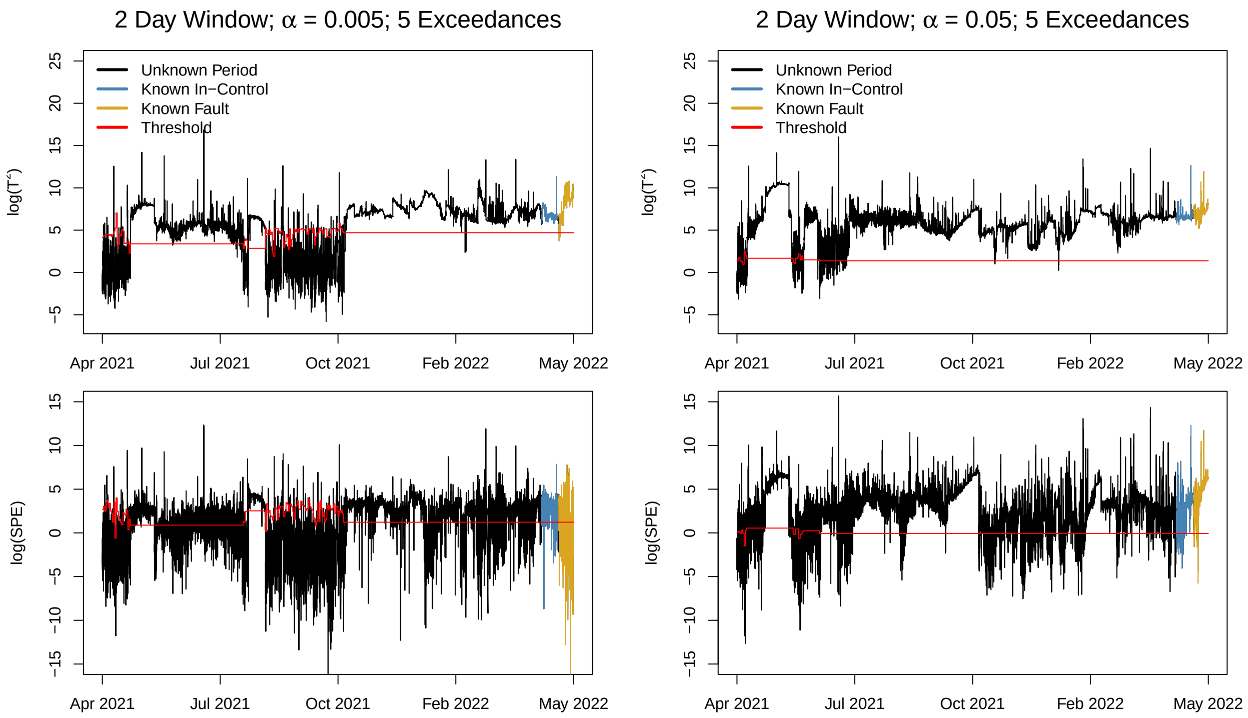

Figure S6: T^2 and SPE with 2 day training window, updating after every 1 day of IC observations using a rejection threshold of $\alpha = 0.005$ (left) or $\alpha = 0.05$ (right). Alarms are only issued after 5 or more consecutive threshold exceedances in either T^2 or SPE.

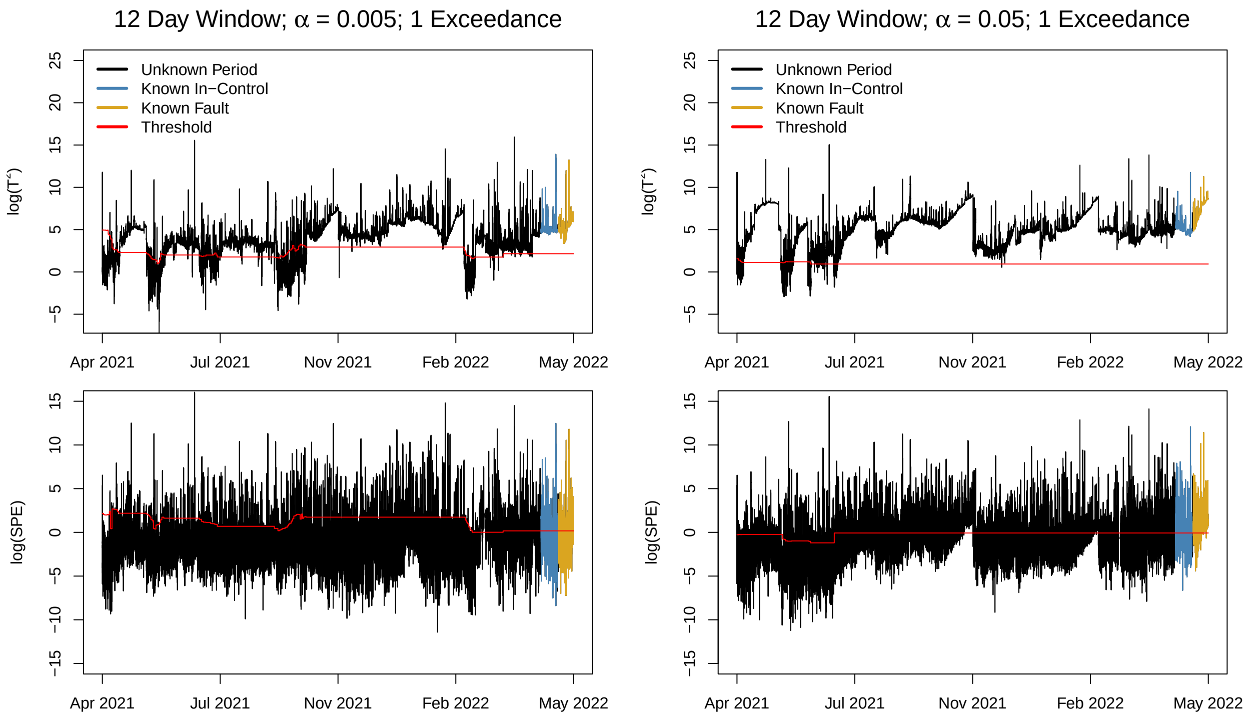

Figure S7: T^2 and SPE with 12 day training window, updating after every 1 day of IC observations using a rejection threshold of $\alpha = 0.005$ (left) and $\alpha = 0.05$ (right).

Table S2: Percent of days retrained during each period for each window size, level of α, and number of exceedances in a row to trigger an alarm. The number of days (d) during each period is also given, and entries in the table show the number of retrained days in parentheses to the right of the percentage.

Window Size (Days)	α	Alarm (# of obs.)	Unknown Period (374 d or 384 d)	Known IC (14 d)	Known Fault (12 d)
2	0.005	1	2.1 (8)	0 (0)	0 (0)
		5	25.3 (97)	0 (0)	0 (0)
	0.05	1	0.5 (2)	0 (0)	0 (0)
		5	4.4 (17)	0 (0)	0 (0)
12	0.005	1	19.5 (73)	0 (0)	0 (0)
		5	74.9 (280)	78.6 (11)	33.3 (4)
	0.05	1	3.5 (13)	0 (0)	0 (0)
		5	16.8 (63)	0 (0)	8.3 (1)
